# Erythroid Adhesion Molecules in Sickle Cell Anaemia Infants: Insights Into Early Pathophysiology

**DOI:** 10.1016/j.ebiom.2014.12.006

**Published:** 2014-12-18

**Authors:** Valentine Brousse, Yves Colin, Catia Pereira, Cecile Arnaud, Marie Helene Odièvre, Anne Boutemy, Corinne Guitton, Mariane de Montalembert, Claudine Lapouméroulie, Julien Picot, Caroline Le Van Kim, Wassim El Nemer

**Affiliations:** a Reference Centre for Sickle Cell Disease, Pediatric Department, Hôpital Universitaire Necker Enfants Malades, APHP, Paris, France; b Université Paris Descartes, Paris, France; c INSERM, U1134, F-75739 Paris, France; d Université Paris Diderot, Sorbonne Paris Cité, UMR_S 1134, F-75739 Paris, France; e Institut National de la Transfusion Sanguine, F-75739 Paris, France; f Laboratoire d'Excellence GR-Ex, France; g Reference Centre for Sickle Cell Disease, Pediatric Department, Centre Hospitalier Intercommunal de Créteil, Créteil, France; h Reference Centre for Sickle Cell Disease, Pediatric Department, Hôpital Louis Mourier, APHP, Colombes, France; i Pediatric Department, Centre Hospitalier Intercommunal Poissy-Saint-Germain-en-Laye, France; j Reference Centre for Sickle Cell Disease, Pediatric Department, Centre Hospitalier Universitaire du Kremlin Bicêtre, APHP, Le Kremlin Bicêtre, France

**Keywords:** Sickle cell anaemia, Red blood cell, Adhesion molecules, Infants, HbF

## Abstract

Sickle cell anaemia (SCA) results from a single mutation in the β globin gene. It is seldom symptomatic in the first semester of life. We analysed the expression pattern of 9 adhesion molecules on red blood cells, in a cohort of 54 SCA and 17 non-SCA very young infants of comparable age (median 144 days, 81–196). Haemoglobin F (HbF) level was unsurprisingly elevated in SCA infants (41.2% ± 11.2) and 2–4 fold higher than in non-SCA infants, yet SCA infants presented significantly decreased Hb level and increased reticulocytosis. Cytometry analysis evidenced a specific expression profile on reticulocytes of SCA infants, with notably an increased expression of the adhesion molecules Lu/BCAM, ICAM-4 and LFA-3, both in percentage of positive cells and in surface density. No significant difference was found on mature red cells. Our findings demonstrate the very early onset of reticulocyte membrane modifications in SCA asymptomatic infants and allow an insight into the first pathological changes with the release of stress reticulocytes expressing a distinctive profile of adhesion molecules.

## Highlights

• Haemolytic anaemia is present in the first semester of life in asymptomatic infants with sickle cell anaemia.
• Reticulocyte membrane modifications and stress reticulocytosis occur even when HbF production is still high.
• Reticulocytes of SCA infants display a specific profile with notably an increased expression of the adhesion molecules Lu/BCAM, ICAM-4 and LFA-3.

## 1 Introduction

Sickle cell anaemia (SCA) is caused by a mutation in the β globin gene. Sickle haemoglobin (HbS) polymerises when deoxygenated, resulting in red cell membrane rigidity and surface protein modifications that subsequently contribute to vaso-occlusion. SCA is seldom symptomatic in the first six months of life. One main explanation lies in the sustained level of foetal haemoglobin (HbF) and F cells during this period (Maier-Redelsperger et al., 1994) preventing HbS polymerisation (Nagel et al., 1979). However, infra clinical vaso occlusion, particularly in the spleen, occurs at a very early age (Rogers et al., 2011) and the absolute reticulocyte count is already elevated in the first semester of life, arguing for the very early onset of haemolysis despite high HbF levels (Meier et al., 2013, Meier et al., 2014). Furthermore, increased evidence supports that HbF elevation during hydroxycarbamide therapy is insufficient to explain the drug's beneficial effect (Segel et al., 2011). In fact, it is now considered that abnormal red blood cell (RBC) adhesiveness in SCA through activation, sustained or increased expression of adhesion molecules is pivotal in the genesis of vaso occlusive crisis, the hallmark of SCA (Hebbel et al., 1980). In this study, we analysed the expression pattern of 9 adhesion molecules on both reticulocytes and mature RBCs in SCA and non-SCA very young infants. These markers are known surface molecules, which allow characterisation of erythroid maturation and/or which are adhesion molecules demonstrated to play an important pathophysiological role (Cartron and Elion, 2008). Our objectives were to gain insight into very early pathophysiology by evidencing distinct profiles specifically attributable to SCA.

## 2 Material and Methods

### 2.1 Patients

Infants diagnosed with SS or S beta° genotypes following neonatal screening were enrolled in a multi-centre prospective study on prognostic factors in SCA (ClinicalTrials.gov: NCT01207037) between September 2010 and March 2013. The institutional review boards of all participating centres approved the study. Written informed consent in accordance with the Declaration of Helsinki was obtained from all parents. Blood sampling was performed at enrolment (3–6 months) at steady state, in asymptomatic infants. In parallel, blood samples from infants with no haemoglobinopathy (non-SCA-infants) were collected.

For each patient complete blood counts, erythrocyte indices were determined using an Advia 120 Hematology System (SIEMENS, Germany).

HbF was quantified by high performance ion-exchange liquid chromatography (HPLC) procedure (BioRad Laboratories, California, USA).

Samples were stored at − 196 °C as previously described (Cartron and Elion, 2008) at the Centre National de Référence pour les Groupes Sanguins, Paris, for secondary flow cytometry analysis.

### 2.2 Flow Cytometry

Flow cytometry analysis was performed using murine monoclonal antibodies against the adhesion molecules CD36, CD44, CD47, CD49d, CD58, CD99, CD147, CD239 and CD242. A BD FACScanto II flow cytometer with HTS (Becton-Dickinson) and FACSDiva software (v6.1.3) were used for acquisition and analysis. The percentage of RBCs expressing each marker and the corresponding mean fluorescence intensity (MFI) were determined under the same conditions for all samples. The percentage of reticulocytes was determined using thiazole orange dye (Retic-CountTM, Becton-Dickinson, San Jose, CA, USA) according to the manufacturer instructions. A total of 10,000 events was analysed for each patient and each molecule.

### 2.3 Statistical Analysis

Results are presented as means or medians ± SD. Statistical analysis was performed with GraphPad Prism 6 (GraphPad Software, La Jolla, CA, USA) using Mann–Whitney test. A difference between two groups was considered statistically significant when *P* < 0.05.

## 3 Results

### 3.1 Asymptomatic SCA Infants Show Early Haemolytic Anaemia Despite Sustained HbF Level (Table 1)

Fifty-four SCA infants were analysed and compared to 17 non-SCA infants. Median age in the two groups was not statistically different (144 days, range 81–196 versus 128, range 68–621, *P* = 0.84) so that no difference in subsequent results would be attributable to this age parameter. Of note and as expected, none of the SCA infants had clinical symptoms related to the disease.

Mean HbF level in SCA infants was 41.2% (± 11.2), a value 2–4 fold higher than reference values (10.4% ± 1.8) (Maier-Redelsperger et al., 1994, Arceci IMH and Smith, 2006), and statistically increased when compared to non-SCA infants (5.7% ± 3.6, *P* < 0.0001).

Median Hb level in SCA infants was 9.1 g/dL (6.5–12), a value significantly decreased in comparison with non-SCA infants (11 g/dL, 7.2–12.6, *P* < 0.001) whilst median reticulocyte percentage was increased in SCA infants (2.9%, 0.5–10 vs 2%, 0.5–4.2, *P* = 0.04).

### 3.2 Flow Cytometry Analysis of SCA Reticulocytes Demonstrates Increased Expression of Erythroid Adhesion Markers

**Fig. 1:**
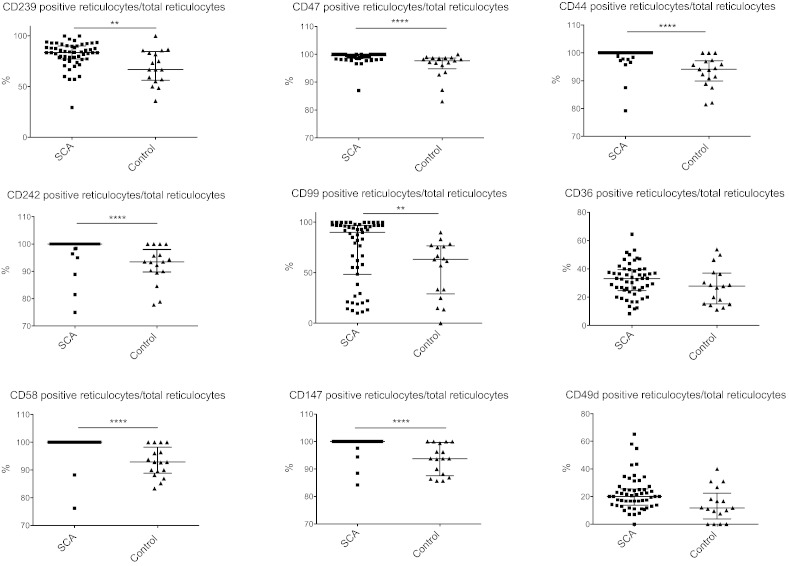
Percentage of positive reticulocytes/total reticulocytes expressing CD239, CD242, CD58, CD47, CD99, CD147, CD44, CD36 and CD49d. Medians (line is at median with interquartile range) were compared using Mann–Whitney test. ****: *P* < 0.0001, **: *P* < 0.01.

Reticulocytes from SCA infants displayed an overall statistically increased expression of the following surface markers: CD239 (Lu/BCAM), CD242 (ICAM-4/LW), CD58 (LFA-3), CD47 (IAP), CD99 (MIC2), CD147 (Basigin) and CD44 (Fig. 1). Because we calculated a ratio of positive reticulocytes on total reticulocytes, these findings are not related to the globally increased percentage of reticulocytes in SCA infants. Conversely, no significant differences were found on mature RBCs between SCA and non-SCA patients (data not shown).

### 3.3 Lu/BCAM, ICAM-4 and LFA-3 are Overexpressed on SCA Reticulocytes

**Fig. 2:**
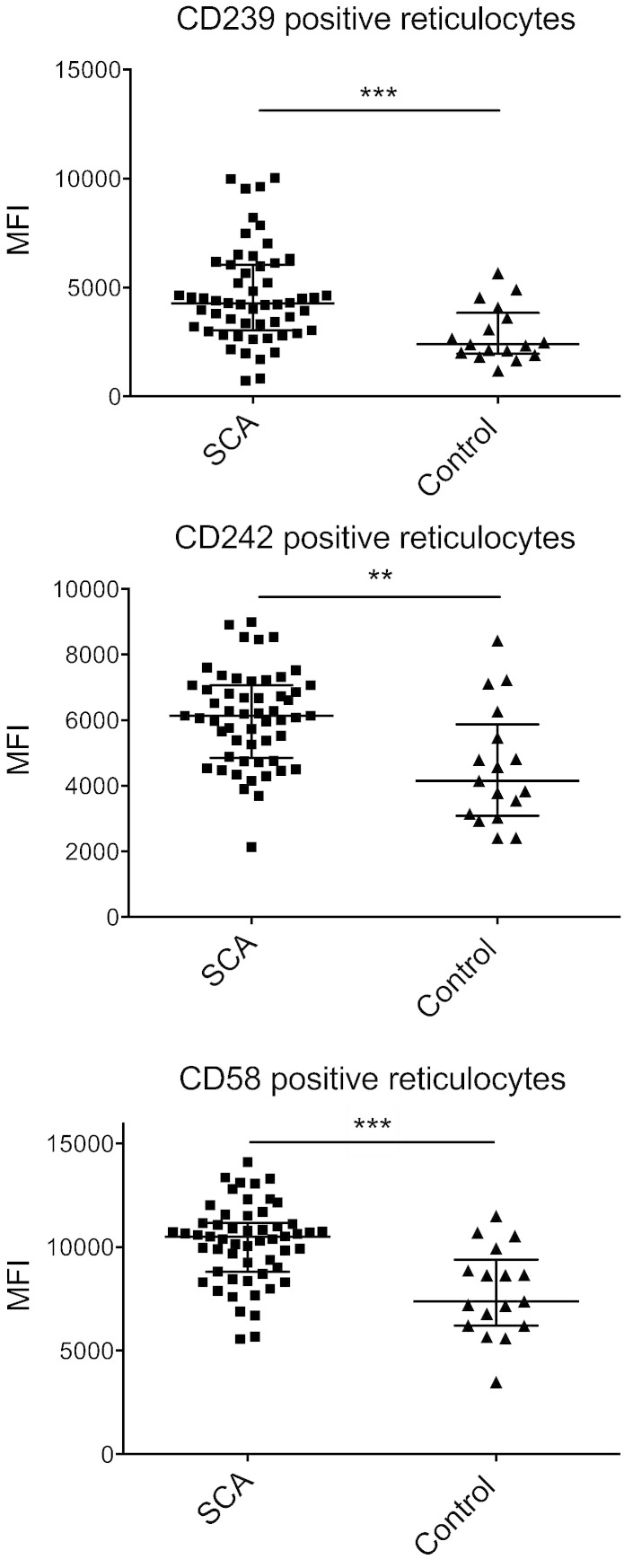
Mean fluorescence intensity of positive reticulocytes/total reticulocytes expressing CD239, CD242 and CD58. Medians (line is at median with interquartile range) were compared using Mann–Whitney test. ***: *P* < 0.001, **: *P* < 0.01.

To further characterise the reticulocyte population in terms of surface molecule expression, we analysed the protein expression level of the 9 surface markers by measuring their mean fluorescence intensity (MFI). Here, MFI was statistically increased concerning two erythroid adhesion molecules involved in SCA pathophysiology, namely Lu/BCAM and ICAM-4, and a poorly described molecule in RBC physiology: LFA-3 (Fig. 2).

## 4 Discussion

Translational research is limited in SCA very young infants either because diagnosis is delayed or because, in case of neonatal diagnosis, parental approval for clinical trials is extremely difficult to obtain at a very young age in the setting of such a severe disease. Here, we confirm in a larger cohort of SCA infants aged less than 6 months, the very early onset of haemolytic anaemia contrasting with the slow decline of HbF. These results, consistent with previous reports (Maier-Redelsperger et al., 1994, Steinberg et al., 2014), illustrate the pitfall of considering elevated HbF level as protective. Measuring global HbF level by HPLC or enumerating F cells overlooks the content of HbF at a cellular level because the number of F-cells with polymer-inhibiting concentrations of HbF is a more important determinant than the concentration of HbF in the hemolysate or the total number of F-cells. However, quantitative methods for measuring the amount of HbF in each F-cell (HbF/F-cell) and plotting the distribution of HbF among F-cells are not available (Steinberg et al., 2014). Measuring HbF does not address therefore the heterogeneous distribution of HbF within RBCs and hence subpopulations of RBCs prone to polymerisation, sickling and haemolysis because of their very low content in HbF. These numerically small subpopulations may in fact play an important pathophysiological role.

Erythroid adhesion molecules play a role in normal red blood cell (RBC) physiology during erythropoiesis and erythrophagocytosis. Expression level, clustering and activation state are critical for the adhesive function. In normal conditions, circulating RBCs are not supposed to adhere to any other cells nor to extracellular matrix components. Most molecules therefore display a decreasing level of expression throughout erythropoiesis. Our data demonstrates that between 3 and 5 months of age, reticulocytes characterised by markers that are otherwise lost or decreased upon maturation, are found in the circulation. At this same time point, mature RBCs display no significant differences compared to non-SCA, consistent with our hypothesis that their release occurred shortly after birth at a time when no dyserythropoiesis is expected to occur, as the HbF switch has not begun. This time frame, therefore, allows a snapshot of the first pathological changes occurring in SCA: the release of reticulocytes with a distinctive profile of stress reticulocytes.

Cytometric analysis of SCA RBCs indeed evidenced reticulocytes overexpressing the erythroid adhesion molecules: Lu/BCAM, ICAM-4 and LFA-3, whilst no difference was evidenced on mature red cells. Overexpression of Lu/BCAM and ICAM-4 has been demonstrated in SCA (El Nemer et al., 1998, Parsons et al., 1999) and, importantly, functional activation by phosphorylation (Gauthier et al., 2005) results in increased RBC adhesion to vascular endothelium and resistance to high shear-stress forces, thereby contributing to VOC genesis (Kaul et al., 2006). Lu/BCAM is the carrier of the Lutheran and the BCAM antigens and the unique erythroid receptor for laminin α5 chain, a major component of the sub endothelial extracellular matrix. It is expressed both on erythroid and endothelial cells and is a receptor for α4β1 integrin expressed on sickle reticulocytes and leukocytes (El Nemer et al., 2007, Bartolucci et al., 2010).

ICAM-4 carries the antigens of the Landsteiner–Wiener (LW) blood group system and is a ligand for a large repertoire of integrins including αVβ3 present on endothelial cells. ICAM-4 undergoes phosphorylation on serine along the PKA-dependent pathway. In addition to its adhesive interaction with endothelial cells, ICAM-4 was shown to play a critical role in vaso-occlusion in murine models through its interaction with αVβ3 (Delahunty et al., 2006). Conversely, no data is available on the role of LFA-3 in erythroid cells, but growing evidence points to LFA-3 as a novel functional marker involved in regulating the self-renewal of tumour-initiating cells in colorectal cancer (Xu et al., 2014). Interestingly, LFA-3 was found on the surface of exosomes released by rat reticulocytes pointing out a mechanism through which it is down regulated during terminal erythroid maturation (Vidal, 2010).

Known adhesion markers previously identified on SCA reticulocytes i.e., CD36 and CD49d (α4β1/Very Late Antigen-4) (Swerlick et al., 1993, Joneckis et al., 1993) were not significantly increased in our SCA infant cohort (Fig. 1). This discrepancy may be explained by the overall relatively low percentage of reticulocytes, as opposed to adults, and is consistent with the findings of Odièvre et al. in SCA children (Odievre et al., 2008).

Recent publications have pointed to the significance of an elevated reticulocyte count in association with disease severity (Meier et al., 2013, Meier et al., 2014). In this study we confirm the very early onset of reticulocytosis in SCA infants and demonstrate the presence of circulating stress reticulocytes with a specific adhesion molecule profile, overexpressing the adhesion molecules Lu/BCAM, ICAM-4 and LFA-3. This numerically small subpopulation of reticulocytes expressing Lu/BCAM, ICAM-4 and LFA-3 could play a role in early infancy notably in splenic injury as these cells would be prone to splenic trapping in the open circulation of the filtering beds, through adhesion to laminin and/or the spleen-specific endothelial cells of the venous sinus (Brousse et al., 2014). Ongoing longitudinal analysis will confirm if this idiosyncratic erythroid adhesion molecule profile in infants indeed correlates with increased haemolysis and adhesion, functional activation and disease severity.

## Funding

This study was funded by a research grant from the French Ministry of Health (PHRC 2007, P071228) and sponsored by the Département de la Recherche Clinique et du Développement de l'Assistance Publique–Hôpitaux de Paris. The Inserm unit 1134 and Institut National de la Transfusion Sanguine benefited from a Région Ile-de-France institutional funding (SESAME 2007 no. F-08-1104/R). The study was supported by grants from Laboratory of Excellence GR-Ex, reference ANR-11-LABX-0051. The labex GR-Ex is funded by the program “Investissements d'avenir” of the French National Research Agency, reference ANR-11-IDEX-0005-02.

None of the funders had any role in study design, data collection, data analysis, interpretation, or writing of the report.

## Authorship Contribution

VB is the principal investigator of the clinical study. VB and WEN designed the study, analysed data, wrote and edited the manuscript. CLVK, YC designed the study, wrote and edited the manuscript. CP, JP and CL performed the cytometry analysis. VB, CA, MHO, AB, CG and MdM included and provided care to the patients.

All authors approved the manuscript.

## Disclosure of Conflicts of Interest

The authors declare no conflict of interest except MdM who is a member of Novartis Speaker's Bureau.

## Figures and Tables

**Table 1:** Patient characteristics.

	SCA	Control	*P*
n = 54	n = 17
Age, days (median, range)	144 (81–196)	128 (68–621)	0.84
Haemoglobin, g/dL (median, range)	9.1 (6.5–12)	11 (7.2–12.6)	< 0.0001
Reticulocytes, % (median, range)	2.9 (0.5–10)	2 (0.5–4.2)	0.04
